# Antifungal Activity and Acute and Repeated-Dose Toxicity Study of Geranyl Cinnamate Ester in Mice

**DOI:** 10.1155/2021/3493625

**Published:** 2021-10-06

**Authors:** Micheli Zanetti, Mikaela Scatolin, Amanda Rebonatto Oltramari, Maria Luiza Lima da Costa Lopes, Rubieli Carla Frezza Zeferino, Gustavo Lopes Colpani, Liz Girardi Müller, Débora de Oliveira, Marcio Antônio Fiori

**Affiliations:** ^1^Department of Chemical Engineering and Food Engineering, Community University of Chapecó Region (UNOCHAPECÓ), Chapecó 89809-000, SC, Brazil; ^2^Graduate Program in Technology and Management of the Innovation, Community University of Chapecó Region (UNOCHAPECÓ), Chapecó 89809 000, SC, Brazil; ^3^Department of Pharmacy, Community University of Chapecó Region (UNOCHAPECÓ), Chapecó 89809-000, SC, Brazil; ^4^Department of Veterinary Medicine, Community University of Chapecó Region (UNOCHAPECÓ), Chapecó 89809-000, SC, Brazil; ^5^Graduate Program in Environmental Science, Community University of Chapecó Region (UNOCHAPECÓ), Chapecó 89809-000, SC, Brazil; ^6^Department of Chemical Engineering and Food Engineering, Federal University of Santa Catarina (UFSC), Florianópolis 88040-900, SC, Brazil

## Abstract

In the present study, the antifungal activity and toxicity of the geranyl cinnamate ester (GCE) were investigated. The GCE showed antifungal activity at a minimum concentration of 0.16 *μ*L/mL against *Candida albicans* and at concentrations greater than 2.5 *μ*L/mL against *Aspergillus niger*. In acute toxicity studies, the administration of GCE (2.000 mg/kg) affected the body weight gain and food intake but did not induce the mortality of the animals studied. After the investigation of repeated-dose toxicity of GCE at 2 and 4 mg/kg, the hematological and biochemical parameters were changed. In addition, the adrenal weight of male mice treated with GCE at 4 mg/kg was affected. In conclusion, according to the Organization for Economic Cooperation and Development (OECD) acute toxicity parameters, the geranyl cinnamate ester can be classified into safety category number 5. The results of this study suggested that the geranyl cinnamate ester may be a source of natural antifungals.

## 1. Introduction

Natural antimicrobial compounds have been used by the food industry as preservatives in industrialized foods. These compounds control and reduce the growth of bacteria and fungi [1, 2]. However, many studies indicate that some natural compounds can cause cancer and allergies in humans [3]. Despite health hazards, these compounds are essential in prolonging the storage time of food, and the use of natural antimicrobials is an attractive opportunity for food preservation.

Natural antimicrobials can be obtained from different sources, including plants, animals, bacteria, algae, and fungi. Several studies with antimicrobial compounds obtained from plants have demonstrated their efficacy when applied for food preservation [4, 5]. In this sense, several studies have evaluated the efficacy of essential oils derived from aromatic plants, such as thymol, carvacrol, allicin, geraniol, limonene, among others, which showed inhibitory activity on the growth of pathogenic bacteria of food origin [6].

Geraniol is an acyclic monoterpene alcohol and is primarily extracted from different essential oils, namely, palmarosa, ninde, and rose oils [7, 8]. In particular, geraniol has a high capacity to inhibit and kill Gram-positive and Gram-negative bacteria, as well as some types of fungi and some types of yeast [8]. The antifungal activity of geraniol is also cited. In the study of Frias and Kozusny-Andreani [9], essential oils extracted from lemon and citronella were tested in four pathogenic fungi (*Candida albicans*, *Nannizzia gypsea*, *Sporothrix schenckii*, and *Aspergillus niger*) and showed high antifungal activity. In addition, in a study by Tang et al. [10], the compounds geraniol and citral showed excellent antifungal effects against common grain pathogens, such as *Aspergillus flavus* and *Aspergillus ochraceus*, in in vitro and in situ tests.

Cinnamic acid, also known as 3-phenyl-2-propenoic acid, consists of a naturally occurring aromatic fatty acid originated from higher plants and found in Estoraques, cinnamon oils, and coca leaves, has low toxicity and a broad spectrum of biological activities against numerous microorganisms. Cinnamic acid is the main constituent of clove oil, which constitutes of approximately 70 to 80% followed by eugenol (4 to 7%) [11].

However, some essential oils are volatile, unstable to light and heat, and easily decomposed depending on the antimicrobial application. Generally, the esterification of some essential oils improves specific substrate properties such as emulsification, dispersion, and overall quality of the consumer products. In this sense, the microbiological and toxicity study of the geranyl cinnamate ester, which was obtained by the esterification reaction between geraniol and cinnamic acid, becomes interesting.

In the literature, no research presents the antifungal activity of the geranyl cinnamate ester. There are works that have studied the antibacterial activity of other esters such as eugenyl acetate against Gram-positive *Staphylococcus aureus* (ATCC 9763) and *Listeria monocytogenes* (ATCC 15117) and Gram-negative bacteria *Escherichia coli* (ATCC 25922) and *Pseudomonas aeruginosa* (ATCC 27853) [12].

There are no reports evaluating the toxicological effects of geranyl cinnamate ester (GCE) and its antimicrobial effect against the fungi *Candida albicans* and *Aspergillus niger*. Thus, the aim of the present study is to investigate the antifungal activity of geranyl cinnamate ester and the toxicity of acute and repeated doses (28 days) in mice.

## 2. Materials and Methods

### 2.1. Geranyl Cinnamate Ester Production

The preparation of the geranyl cinnamate ester was carried out according to Zanetti et al. [13] by the esterification reaction of cinnamic acid with geraniol (≥99%) from Sigma-Aldrich (Brazil), under the following reaction conditions: 70°C, 15 wt% of immobilized *Candida Antarctica* NS88011, and 3 : 1 geraniol to cinnamic acid molar ratio. The reaction was carried out in Erlenmeyer glass flasks (250 mL) using an orbital shaker (150 rpm), 10 mL n-heptane as solvent, and 2 h reaction time for all experiments. To purify the geranyl cinnamate ester and to remove the unreacted reagents (enzyme *Candida Antarctica*, cinnamic acid, and geraniol) after all experiments, the final product was filtered with membranes and then evaporated on a rotary evaporator with a maximum temperature of 40°C. For this reaction, a 97% conversion of geraniol to geranyl cinnamate ester was obtained.

### 2.2. Experimental Design

#### 2.2.1. Antifungal Activity of Geranyl Cinnamate Ester

Antifungal activity of the geranyl cinnamate ester was evaluated according to the antifungal susceptibility testing method described by the NCCLS (2004), with adaptations and with two genera of fungi: yeast *Candida albicans* (ATCC 24433) and *Aspergillus niger* (ATCC 16888). The strains of *Candida albicans* and *Aspergillus niger* were obtained from the Laboratory of Mycology of the Community University of the Region of Chapecó—Unochapecó.

The strains were reactivated with Sabouraud dextrose broth, and for the study, the fungal suspensions were prepared by choosing five colonies with a diameter of approximately 1 mm after incubation of 24 h of the *Candida* species. The colonies were suspended in 5 mL of sterile saline (0.90% saline), and the resultant suspension was homogenized on a vortex shaker for 15 seconds. Subsequently, a saline solution was added to obtain the turbidity equivalent to the standard solution of the McFarland 0.5 scale to obtain a standard yeast suspension containing approximately 10^5^ microorganisms per 1 mL.

Assays were performed using 20 mL of sterile Sabouraud dextrose agar culture medium at 65°C in Petri dishes (50 × 10 mm) and were allowed to solidify. Different amounts of geranyl cinnamate were added to the agar in different plates, obtaining different concentrations as shown in Table 1.

A volume of 10 *μ*L of the microorganism suspension was then inoculated onto the agar and spread with the aid of a Drigalski loop over the entire surface of the plate. The plates were incubated at 36 ± 1°C in a greenhouse (J Prolab, model B3) for 48 h for the *Candida albicans* and for 5 days at 27 ± 1°C for Aspergillus *niger*. After this time, antifungal activities were evaluated by the presence or absence of colony formation. To verify the growth of the microorganisms, a control plate was prepared with the microorganism without the addition of antifungal compounds.

#### 2.2.2. In Vivo Assays

*(1) Animals*. Male and female (nulliparous and nonpregnant) mice (20–30 g) from Unochapecó bioterium (Chapecó-SC) were used. Animals were housed in groups of five mice in plastic cages (28.0 × 12.5 × 19.0 cm) at constant room temperature (22 ± 2°C) and humidity (40–60%), under a 12 h light/dark cycle with free access to food (Biobase®) and water *ad libitum*. Experiments were approved by Animal Care Local Ethical Committee (CEUA-UNOCHAPECÓ; Protocol 008/2018). Animal care and experiments were conducted in accordance with Brazilian law (Brazil, 2008; CONCEA, 2018) and EU Directive 2010/63/EU for animal experiments.

*(2) Treatments*. Geranyl cinnamate ester (GCE) is a water-insoluble compound and, therefore, it was dissolved in corn oil (0.1, 0.2, 0.4, or 200.0 mg/mL), according to the OECD Guidelines 423 [14] and 407 [15] and was administered by gavage. In the acute toxicity studies, the GCE was orally administered to mice at a dosage of 2000.0 mg/kg and for the repeated-dose toxicity studies at 1.0, 2.0, or 4.0 mg/kg (p.o.) for 28 days. All treatments were with respect to the dosage of 10 mL/kg body weight. To determine the concentration to be used in the tests, the maximum percentages of addition of active compounds in packaging (4%) and the minimum amount of ester necessary to inhibit microbial growth were considered. It was considered for the calculation of 100% release of the active compound from the packaging to the product and, thus, a value of 1 mg/kg is obtained. The euthanasia was performed with thiopental sodium (50 mg/kg, i.p.) preceded by hydrochloride lidocaine (10 mg/kg, i.p.).

*(3) Toxicity Studies*. The toxicity studies were based on the guidelines of the Organization for Economic Cooperation and Development (OECD). The acute oral toxicity studies were performed according to Guideline 423 [14], and the repeated-dose (28-day oral administration) toxicity studies followed the Guideline 407 [15]. These OECD guidelines are the worldwide reference for chemical testing.

Regarding the acute toxicity test (OECD 423), female mice received a single GCE dose (2000 mg/kg, p.o.) (*n* = 3 mice/experimental step). The animals were fasted before administration (food but not water was withheld for 3 h). After the administration, animals were observed with special attention during the first 4 hours and 12 hours later, and every day for 14 days. The body weight gain and food intake were registered every two days during the experimental period. Observations of the abnormal behavior of female mice such as piloerection, palpebral ptosis, abdominal writhing, muscular tonus, motor activity, hypothermia, shacking, posterior paw paralysis, salivation, bronchial secretion, lethargy, diarrhea, and convulsions were considered. Moreover, the number of deaths was registered.

For repeated-dose toxicity tests (OECD 407), the GCE was administered in three different doses by gavage once a day for 28 days. Female (*n* = 20) and male (*n* = 20) mice were divided into four groups containing five animals by gender, according to the OECD Guideline 407 [15]: group I: control, treated with vehicle (corn oil, 10.0 mL/kg); group II: GCE 1.0 mg/kg, p.o.; group III: GCE 2.0 mg/kg, p.o.; and group IV: GCE 4.0 mg/kg, p.o. The 1.0 mg/kg dose was chosen according to the profile of GCE release from the package to food. Considering that the preservative would be ingested at 1.0 mg/kg, the doses of 2.0 and 4.0 mg/kg were defined in accordance to the OECD 407 (2 to 4 fold intervals for setting the dose levels). The same toxicity signs described in the OECD 423 were observed. Food intake and body weight gain were registered every two days throughout the experiment.

The euthanasia of animals occurred at the end of the experimental protocols on the 15th and 29th days (acute and repeated-dose toxicity study, respectively). Mice were euthanized with thiopental sodium (50.0 mg/kg, i.p.) preceded by lidocaine hydrochloride (10.0 mg/kg, i.p.). Blood and urine were collected from the hepatic portal vein and bladder, respectively, from mice submitted to the repeated-dose toxicity study.

The organs (brain, heart, thymus, spleen, adrenals, kidney, and liver (OECD 2008)) were removed (both after the acute and subacute study) and weighed for statistical analysis and further histopathological studies. The relative organ weight was calculated considering the body weight of the mouse by using the following equation:

(1) $$\begin{array}{c} \text{relative organ weight}\left(\%\right)=\frac{\text{organ weight}*100}{\text{mouse body weight}}. \end{array}$$

#### 2.2.3. Biochemical Parameters

Serum investigations were made for sodium (Na), potassium (K), glucose (GLU), total cholesterol (COL)and fraction (LDL), triglycerides (TRI), uric acid (UAC), creatinine (CRE), total protein (PRO), albumin (ALB), and two enzymes indicative of hepatocellular effects: alanine aminotransferase (ALT) and alkaline phosphatase (AP). The analyses were performed with Labtest® kits using a spectrophotometer BTS-310® (Biosystems®). Considering that the blood volume collected from the mice varied from animal to animal, the final number of animals used in the tests was between 3 and 5 per group.

#### 2.2.4. Hematological Parameters

Blood was collected into EDTA tubes (0.5 mL), and some parameters were evaluated: hemoglobin (Hb), red cell distribution width (RDW), haematocrit (HCT), mean corpuscular volume (MCV), mean cell hemoglobin (MCH), mean cell corpuscular hemoglobin concentration (MCHC), white blood cell counts (WBC), erythrocyte count, reticulocytes, eosinophils (E), monocytes (M), neutrophils (N), lymphocytes (L), and platelet counts. The analyses were performed on the ABX Micros 60® equipment. Considering the fast blood coagulation, the final number of mice haemogram varied between 3 and 5 per group.

#### 2.2.5. Urinalysis

This analysis was performed using Uriquest Plus® (Labtest®, Brazil) semiquantitative fast determination reagent strips for urobilinogen, glucose, ketone bodies, bilirubin, total protein, ascorbic acid, blood, nitrite, leucocytes, pH, and density.

#### 2.2.6. Histopathology

Five animals (two males and three females) from each treatment group were randomly chosen for the histological analysis. Brain, heart, thymus, spleen, adrenals, kidneys, and liver were fixed in neutral buffered 10% formalin. The samples were dehydrated with alcohol, cleared with xylene, embedded in paraffin, sectioned, and stained with hematoxylin and eosin. Samples were then processed and examined by optical microscopy.

### 2.3. Statistical Analysis

Two-way repeated-measures analysis of variance (ANOVA) followed by the Bonferroni test was used to evaluate the relative body weight and food intake of mice. One-way ANOVA *post hoc* Bonferroni was performed for the evaluation of the hematological and biochemical analysis and relative weight of organs (repeated-dose study). The relative weight of organs in the acute toxicity study was analysed with the unpaired *t*-test. GraphPad Prism® 5.0 software was used to perform the statistical analysis. Data were expressed as mean ± SEM. The level of significance was set as *p* < 0.05.

## 3. Results

### 3.1. Results Obtained for the Antifungal Activity of the Geranyl Cinnamate Ester

**Figure 1:**
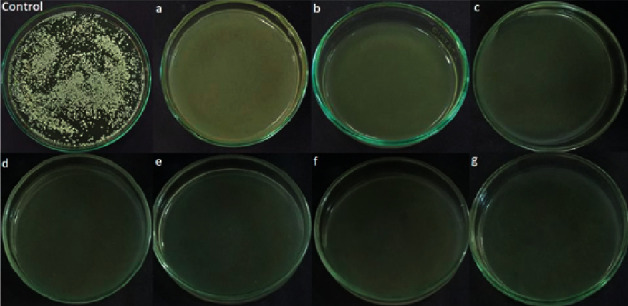
Results of the agar diffusion assays for the antifungal activity of geranyl cinnamate ester with yeast *Candida albicans* of different concentrations: (a) 10.00 *μ*L/mL, (b) 5.00 *μ*L/mL, (c) 2.50 *μ*L/mL, (d) 1.25 *μ*L/mL, (e) 0.62 *μ*L/mL, (f) 0.31 *μ*L/mL, and (g) 0.16 *μ*L/mL.

Antifungal activity of the geranyl cinnamate ester was carried out with yeast *Candida albicans*. The tests were performed with seven different concentrations of geranyl cinnamate ester, and the diameter values of *Candida albicans* growth inhibitor halos were compared with the control sample, which contained only pure agar without the active compound. The results are presented in Figure 1.

Geranyl cinnamate ester was active for all concentrations tested, and the minimum concentration was 0.16 *μ*L/mL (letter f) against *Candida albicans*. It is possible to observe that, in the control sample, there was no inhibition of the growth of the fungus.

**Figure 2:**
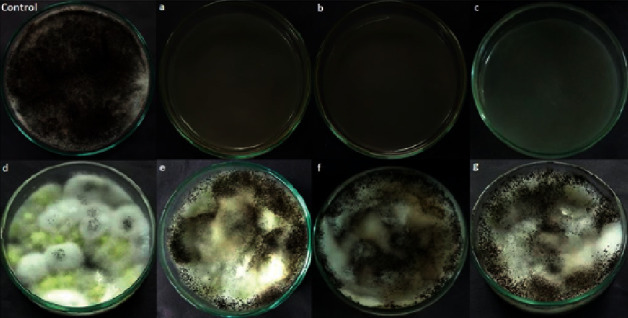
Results of the agar diffusion assays for the antifungal activity of geranyl cinnamate ester with yeast *Aspergillus niger* of different concentrations: (a) 10.00 *μ*L/mL, (b) 5.00 *μ*L/mL, (c) 2.50 *μ*L/mL, (d) 1.25 *μ*L/mL, (e) 0.62 *μ*L/mL, (f) 0.31 *μ*L/mL, and (g) 0.16 *μ*L/mL.

Antifungal activity of the geranyl cinnamate ester was also evaluated against the fungus *Aspergillus niger*. The tests were performed with different concentrations of geranyl cinnamate ester, and the results are shown in Figure 2.

The geranyl cinnamate ester showed antifungal activity against *Aspergillus niger* fungus when used at a concentration of up to 2.5 *μ*L/mL (letter c).

### 3.2. Acute Toxicity

**Figure 3:**
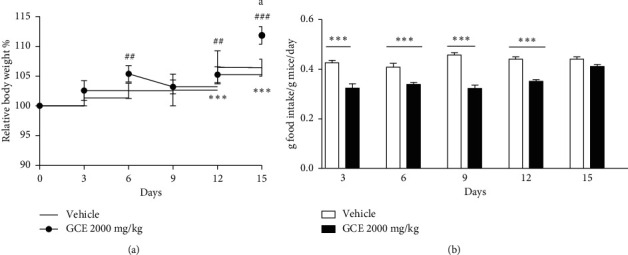
Effect of geranyl cinnamate ester (GCE) acute treatment (2000 mg/kg, p.o.) on relative body weight (%) of female mice (*n* = 3–6 mice/group) (a) and food intake (g food intake/g mice/day) (b). Data are expressed as mean + SEM. Two-way repeated-measures ANOVA *post hoc* Bonferroni. Relative body weight (a): different from the initial weight (day 0). ^*∗∗∗*^*p* < 0.001 (corn oil-treated, 10 mL/kg, p.o.), ^##^*p* < 0.01 (GCE-treated, 2000 mg/kg, p.o.), and ^###^*p* < 0.01 (GCE-treated, 2000 mg/kg, p.o.), and *p* < 0.05 different from the vehicle group (corn oil-treated, 10 mL/kg, p.o.) in the same day of measurement. Food intake (b): ^*∗∗∗*^*p* < 0.001 different from the vehicle group (corn oil-treated, 10 mL/kg, p.o.).

**Figure 4:**
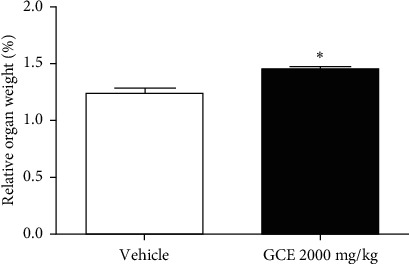
Effect of the geranyl cinnamate ester (GCE) acute treatment (2000 mg/kg, p.o.) on the relative weight of female mice (*n* = 3–6 mice/group) kidney. Data are expressed as mean ± SEM. Unpaired *t*-test: ^*∗*^*p* < 0.05 compared to the vehicle-treated group (corn oil, 10 mL/kg, p.o.).

On the first day of the experiment (during 4 h after administration), GCE-treated mice presented lethargy and sedation, without the loss of reflexes and respiratory depression. No deaths occurred during the experiment. The body weight of the GCE-treated mice increased at the 6th (*p* < 0.01), 12th (*p* < 0.01), and 15th day after treatment (*p* < 0.001). The results indicate a weight gain of the mice treated with corn oil (*p* < 0.001) on the 12th and 15th day, when compared to the beginning (day 0) of the treatment and there was a significant (*p* < 0.05) body weight gain of the GCE-treated mice in comparison with the vehicle-treated group at the 15th day after the oral administration (Figure 3(a)). However, the food intake of the GCE-treated mice was significantly (*p* < 0.001) lower than the control group food intake from the 3rd to the 12th day after the treatment (Figure 3(b)). The oral administration of GCE induced a significant increase (*p* < 0.05) in the relative weight of the kidney when compared to the corn oil-treated mice (Figure 4). There were no significant changes in the relative weight of brain, heart, thymus, liver, and adrenals between the groups (data not shown).

### 3.3. Repeated-Dose Toxicity

Gross behavior of the animals was observed during the 28 days of administration. One male mouse treated with GCE 4 mg/kg group presented diarrhea at the first week of administration. Female mice did not present any sign of toxicity.

#### 3.3.1. Body Weight Gain

**Figure 5:**
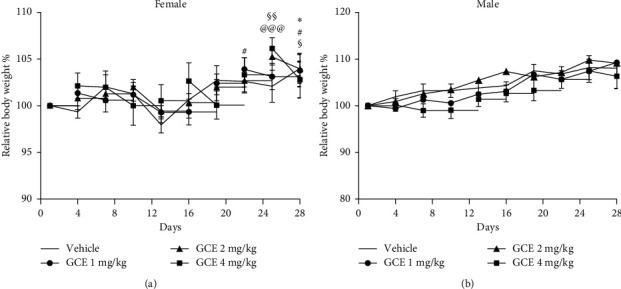
Effect of geranyl cinnamate ester (GCE) repeated-dose treatment (1, 2, and 4 mg/kg, p.o.) on the relative body weight of female (a) and male (b) mice (*n* = 5 mice/group). Data are expressed as mean + SEM. Two-way repeated-measures ANOVA *post hoc* Bonferroni. Symbols represent differences in relation to the first measurement (day 1) in the same treatment group (^*∗*^*p* < 0.05: vehicle group; ^#^*p* < 0.05: GCE 1 mg/kg; ^§§^*p* < 0.01: GCE 2 mg/kg, and ^@@@^*p* < 0.001: GCE 4 mg/kg).

All female mice treated with GCE (1, 2, and 4 mg/kg, p.o.; Figure 5(a)) presented a significant body weight gain when compared to the 1st day of treatment. At the 22nd and 28th day of observation, the body weight of GCE 1 mg/kg-treated female mice was significantly (*p* < 0.05) increased in comparison with the first (day 1) measurement taken in the same group. The body weight of GCE 2 mg/kg-treated female mice was significantly higher at the 25th (*p* < 0.01) and 28th (*p* < 0.05) day when compared to the 1st day of treatment. The body weight of GCE 4 mg/kg-treated female mice was significantly (*p* < 0.001) increased at the 25th day of treatment. The body weight of the vehicle-treated female mice (corn oil, 10 mL/kg) was significantly (*p* < 0.05) increased at the last day (28th) of the experiment when compared to the 1st day of treatment.

The body weight of male mice (Figure 5(b)) was not affected by the GCE administration. There were no significant differences between the weight gain of the GCE-treated groups in comparison with the vehicle group at the same day of treatment.

#### 3.3.2. Food Intake

**Figure 6:**
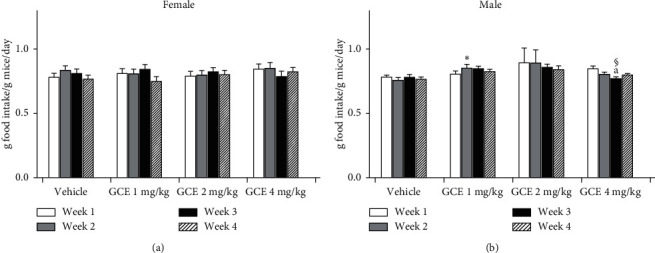
Effect of geranyl cinnamate ester (GCE) repeated-dose treatment (1, 2, and 4 mg/kg, p.o.) on food intake by female (a) and male (b) mice (g food intake/g mice/day). Data are expressed as mean + SEM (*n* = 5 mice/group). Two-way repeated-measures ANOVA *post hoc* Bonferroni. ^*∗*^*p* < 0.05 different from the vehicle group (corn oil-treated, 10 mL/kg, p.o.) in the same week; ^§^*p* < 0.05 different from the GCE 2 mg/kg-treated group in the same week; ^a^p < 0.05 different from the week 1 of the same treatment group.

Figure 3 demonstrates the food intake of the GCE and vehicle-treated female (Figure 6(a)) and male (Figure 6(b)) mice. The food intake of female mice did not present any significant variation between the vehicle group and the GCE-treated groups (Figure 6(a)); while the food intake of the male mice (Figure 6(b)) was significantly affected by the GCE treatment: food consumption of GEC 1 mg/kg-treated group was significantly (*p* < 0.05) higher at week 2 in relation to the vehicle-treated group (corn oil-treated, 10 mg/mL) in the same week. The food consumption of GCE 4 mg/kg-treated group was significantly (*p* < 0.05) decreased at week 3 in relation to the GCE 2 mg/kg-treated group in the same week, and there was a significant (*p* < 0.05) decrease at week 3 in relation to week 1 of the same treatment group.

#### 3.3.3. Haematological Parameters

Haematological data of male and female ECG (1, 2, 4 mg/kg, p.o.) and vehicle-treated mice are presented in Table 2. Mice (female and male) treated with GCE at 4 mg/kg presented a significantly (*p* < 0.05) lower number of reticulocytes (%) when compared to GCE 1 mg/kg-treated group.

Lymphocytes (%) of the GCE 4 mg/kg-treated male mice presented a significant (*p* < 0.05) decrease in lymphocytes when compared to the vehicle-treated group (corn oil, 10 mg/mL). No changes were detected between the female mice groups. GCE 4 mg/kg-treated male mice presented a significant (*p* < 0.05) increase in neutrophils (%) compared to the vehicle-treated group, while no changes were detected between the female mice groups.

Platelets (×10^3^/mm^3^) of the male mice treated with GCE at 4 mg/kg were significantly decreased when compared to the GCE 2 mg/kg-treated group (*p* < 0.01), GCE 1 mg/kg-treated group (*p* < 0.05), and vehicle-treated group (*p* < 0.05). The female mice platelet number was not altered between the groups.

Hemoglobin (Hb), red cell distribution width (RDW), haematocrit (HCT), mean corpuscular volume (MCV), mean cell hemoglobin (MCH), mean cell corpuscular hemoglobin concentration (MCHC), white blood cell counts (WBC), erythrocytes counts, and monocytes did not change significantly between groups.

#### 3.3.4. Biochemical Parameters

Several biochemical parameters were affected by the mice treatment with GCE. These data are depicted in Figure 4 (female mice) and Figure 5 (male mice).

The Na^+^ (mEq/L) serum level in the GCE 1 mg/kg-treated female mice (Figure 4(a)) was significantly (*p* < 0.05) decreased in relation to the vehicle group. No variations in Na^+^ (mEq/L) levels were detected in the serum of male mice (Figure 5(a)).

The K^+^ serum levels (mEq/L) in the female (Figure 4(b)) and male (Figure 5(b)) GCE 4 mg/kg-treated groups were significantly (*p* < 0.05) increased in female mice and decreased in male mice when compared to the GCE 2 mg/kg-treated groups.

Treatment of female mice (Figure 4(c)) with GCE at 4 mg/kg induced a decrease in PRO (g/dL) serum levels when compared to the vehicle-treated animals; GCE 1 mg/kg-treated male mice (Figure 5(c)) presented a significant (*p* < 0.05) increase in PRO levels in relation to the GCE 2 and 4 mg/kg-treated groups.

Female mice that received GCE at 2 and 4 mg/kg (p.o.) showed significantly (*p* < 0.05) decreased ALB (g/dL) serum levels (Figure 4(d)) when compared to the vehicle-treated group, while male mice that were orally treated with GCE at 2 mg/kg presented a significant (*p* < 0.05) decrease in the ALB serum levels (Figure 5(d)) in relation to the vehicle and GCE 1 mg/kg-treated groups.

The treatment of female mice with GCE at the highest dose elicited a significant (*p* < 0.05) increase in GLU (mg/dL) serum levels (Figure 4(e)) in relation to the vehicle-treated group; in male mice, there was a significant (*p* < 0.05) increase in GLU serum levels (Figure 5(e)) of the GCE 2 mg/kg-treated group in comparison with the GCE 1 mg/kg-treated animals.

**Figure 7:**
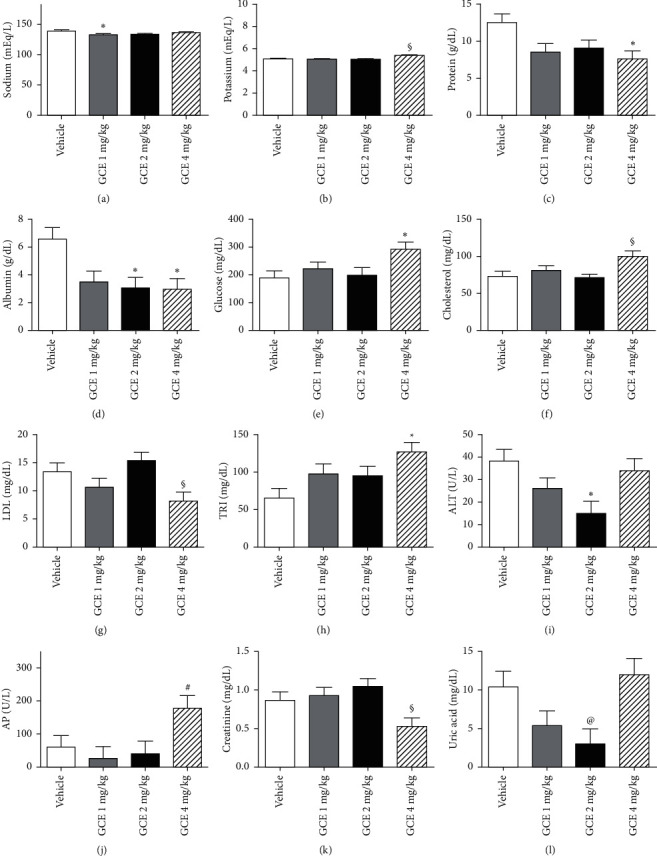
Effect of the geranyl cinnamate ester (GCE) repeated-dose treatment (1, 2 and 4 mg/kg, p.o.) on female mice biochemical parameters. Data are expressed as mean + SEM. One-way ANOVA *post hoc* Bonferroni (*n* = 3–5 animals/group). ^*∗*^*p* < 0.05 different from vehicle group; ^#^*p* < 0.05 different from GCE 1 mg/kg-treated group; ^§^*p* < 0.05 different from GCE 2 mg/kg-treated group; and ^@^*p* < 0.05 different from GCE 4 mg/kg-treated group. (a) Serum sodium (Na); (b) potassium (K); (c) total protein; (d) albumin; (e) glucose; (f) total cholesterol; (g) cholesterol fraction (LDL); (h) triglycerides; (i) alanine aminotransferase (ALT); (j) alkaline phosphatase (AP); (k) creatinine, and (l) uric acid were evaluated.

**Figure 8:**
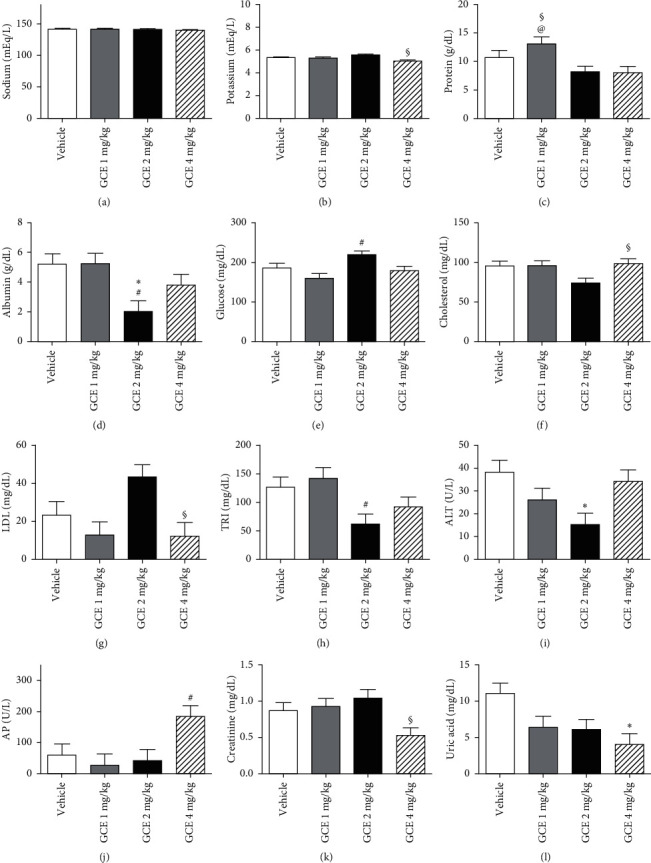
Effect of the geranyl cinnamate ester (GCE) repeated-dose treatment (1, 2, and 4 mg/kg, p.o.) on male mice biochemical parameters. Data are expressed as mean + SEM. One-way ANOVA *post hoc* Bonferroni (*n* = 3–5 animals/group). ^*∗*^*p* < 0.05 different from vehicle group; ^#^*p* < 0.05 different from GCE 1 mg/kg-treated group; ^§^*p* < 0.05 different from GCE 2 mg/kg-treated group; ^@^*p* < 0.05 different from GCE 4 mg/kg-treated group. (a) Serum sodium (Na); (b) potassium (K); (c) total protein; (d) albumin; (e) glucose; (f) total cholesterol; (g) cholesterol fraction (LDL); (h) triglycerides; (i) alanine aminotransferase (ALT); (j) alkaline phosphatase (AP); (k) creatinine and (l) uric acid were evaluated.

COL (mg/dL) levels in the serum of female (Figure 7(f)) and male (Figure 8(f)) GCE 4 mg/kg-treated mice were significantly (*p* < 0.05) increased in relation to the GCE 2 mg/kg-treated group.

LDL serum levels (mg/dL) were affected in the animals that received the highest ECG dose: in both sexes (Figures 4(g) and 5(g)), there was a significant (*p* < 0.05) decrease in comparison with the GCE 2 mg/kg-treated group.

TRI (mg/dL) levels in the serum of female mice (Figure 4(h)) treated with GCE at 4 mg/kg were significantly (*p* < 0.05) increased in relation to the vehicle-treated group; male mice (Figure 5(h)) that received GCE at 2 mg/kg presented a significant (*p* < 0.05) decrease in serum TRI levels in relation to the GCE 1 mg/kg-treated group.

ALT (mg/dL) levels in the serum of female (Figure 4(i)) and male (Figure 5(i)) mice treated with GCE at 2 mg/kg were significantly (*p* < 0.05) decreased in relation to the vehicle-treated group.

Female (Figure 4(j)) and male (Figure 5(j)) mice treated with GCE at 4 mg/kg presented significantly (*p* < 0.05) increased AP (U/L) serum levels when compared to the GCE 1 mg/kg-treated group.

CRE (mg/dL) serum levels of female (Figure 4(k)) and male (Figure 8(k)) GCE 4 mg/kg-treated groups were significantly (*p* < 0.05) decreased in relation to the GCE 2 mg/kg-treated groups.

Treatment of female mice (Figure 4(l)) with GCE at 2 mg/kg significantly (*p* < 0.05) decreased UAC (mg/dL) serum levels in relation to the GCE 4 mg/kg-treated animals; while male (Figure 5(l)) GCE 4 mg/kg-treated mice UAC levels were significantly (*p* < 0.05) decreased in relation to the vehicle-treated group.

#### 3.3.5. Relative Organs' Weights (%)

Data of the relative organs' weight (%) of male and female GCE-treated mice (1, 2, and 4 mg/kg, p.o.) are shown in Table 3. The adrenal gland relative weights of the GCE 4 mg/kg-treated male animals were significantly (*p* < 0.05) increased when compared to the vehicle-treated group (corn oil 10 mg/mL, p.o.). The other organs (liver, kidney, spleen, heart, thymus, and brain) from the ECG-treated male and female mice did not show any significant differences in the relative weight (%) in comparison with the vehicle-treated animals.

#### 3.3.6. Urinalysis

Urinary analysis did not present any significant variation in the parameters evaluated in the groups treated with the GCE when compared to the vehicle-treated group (data not shown).

#### 3.3.7. Histopathological Parameters

Organs from the male and female mice treated during 28 days with GCE did not present significant anatomic or histopathological variations at any dose in comparison with the organs from the vehicle-treated group.

## 4. Discussion

In the present study, it was demonstrated that the geranyl cinnamate ester (GCE) was active for all concentrations tested in the *Candida albicans* and for the *Aspergillus niger* fungus when used with a concentration of up to 2.5 *μ*L/mL. In the scientific literature, there were no studies related to the GCE, only some related to the geraniol activity, but without the optimization of the concentrations in the synthesis reactions. Therefore, the data obtained in this stage of the work confirm the antimicrobial activity of the GCE with the fungi tested, showing the importance that this compound may have in an area where few studies are published for growth control, using natural compounds.

In a study of the antimicrobial activity of an essential oil containing in its composition thymol, carvacrol, and geraniol (that is the compound used to obtain our ester), against the fungus *Candida albicans*, Botelho et al. [16] demonstrated that the essential oil exhibited an antifungal activity. Additionally, in the work of Marcos-Arias et al. [17], the authors reported that geraniol showed antifungal activity against strains of *Candida albicans*. Moreover, in the work carried out by Wang et al. [18], it was observed that geraniol showed antifungal activity against *A. flavus*, *A. carbonarius*, and *P. viridicatum* with values of minimum inhibitory concentration (MIC) above 5.00 *μ*L/mL.

Ternus et al. [8] evaluated the antimicrobial activity of geraniol essential oil against different microorganisms. In the agar diffusion test for *Staphylococcus aureus*, the mean diameter of the zone of inhibition halo was 35.3 ± 0.08 mm and for *Escherichia coli*, the mean diameter of the halo was (25.5 ± 0.05) mm. For cinnamic acid to bacteria of type *Staphylococcus aureus*, the average diameter of the inhibition halo was (16.5 ± 0.10) mm and for the bacteria of the *Escherichia coli* halo average diameter was (11.0 ± 0.06) mm.

The work carried out by Zanetti et al. [13] brings the data on the antimicrobial activity of the geranyl cinnamate ester against the same bacteria, and it is possible to observe that it is bacteria of type *Staphylococcus aureus* the inhibition zone had an average diameter halo of 22.7 ± 0.60 mm and for *Escherichia coli*, the zone of inhibition had an average diameter of 17.2 ± 0.32 mm. These works [8, 13] show that the junction of geraniol with cinnamic acid produced the geranyl cinnamate ester, which is a compound microbiologically very active for the bacteria *Staphylococcus aureus* and *Escherichia coli*.

The mechanism of action of esters against bacteria and fungi is possibly the same as that of essential oils. The constituents of the oils destroy the cytoplasmic membrane and the cell wall of bacteria and fungi. This effect results in the extravasation of the cytoplasm and its coagulation, in addition to inhibiting cellular respiration [19].

This study also presented for the first time the acute and repeated-dose toxicity of GCE. The OECD guidelines 423 and 407 used to perform the toxicity tests are worldwide accepted and considered the standard model to assess the toxicity of chemical compounds [20]. Considering that the GCE is a candidate to be used as an additive for the food industry, our results are considerably relevant.

Acute toxicity study demonstrated that mice treatment with GCE at 2000 mg/kg (p.o.) decreased food intake and did not affect weight gain. Therefore, the decreased food consumption was not sufficient to affect mice weight gain and might be related to the sedation elicited by the GCE administration. Furthermore, GCE evoked an increase in the relative weight of the mice kidneys, suggesting a possible acute toxicity to this organ. Nevertheless, GCE acute treatment did not induce mice death; therefore, this compound is classified in the safety category 5 of the Global Harmonized Classification System (GHS), and its LD50 (median lethal acute dose) is above 2000 mg/kg [14].

Repeated-dose toxicity tests provided data about persistent or cumulative toxic effects on target organs, dose-response relationships, and the no‐observed‐adverse‐effect level (NOAEL) [15], where both sexes of mice were used for the repeated-dose toxicity study, since toxicological studies demonstrated some differences in the sensitivity between females and males [15].

Gross behavior of the female GCE-treated (1, 2, and 4 mg/kg, p.o.) mice groups was considered normal, and no toxicity symptoms were noted during the 28 days of treatment. However, diarrhea was observed at the first week of administration in only one male mice treated with the highest dose of GCE, which could represent an adverse effect of the treatment. Moreover, no animals died during the experimental period, and significant hematological and biochemical variations were observed in animals treated with GCE at the highest doses (2 mg/kg and 4 mg/kg) only.

Treatment of female mice with GCE did not impact the food intake, neither body weight gain, since all groups presented a significant weight gain during the experiment. However, male mice treated with GCE did not present a significant increase in body weight, which could be related to the decreased food consumption elicited by the GCE at 4 mg/kg. Considering that the food intake of the vehicle-treated group did not change along the experimental period, we may infer that the stress caused by the repeated orogastric gavage [21] did not affect the animals' food intake. Therefore, the decrease in food intake of GCE 4 mg/kg-treated male mice could indicate adverse effects [22, 23] or might represent an anorexigenic effect [24] of the compound.

No macroscopic lesions, abnormal anatomic aspects, and no variations in the relative weight of the organs were observed in the female mice after the administration of GCE. Nevertheless, the treatment of male mice with GCE 4 mg/kg elicited an increase in the adrenal glands' relative weight in comparison with the vehicle group. Adrenal hyperplasia may be related to hypersecretion of corticosterone by the adrenal cortex as a consequence of adenohypophysis stimulation, which secrets a large amount of ACTH (adrenocorticotropic hormone) [25]. Therefore, we may speculate that the GCE at the highest dose could cause a dysfunction in the hypothalamic-pituitary-adrenal (HPA) axis in male mice.

This hypothesis is consonant to the neutrophilia and lymphopenia found in male mice treated with GCE 4 mg/kg. These abnormalities characterize a corticoid-mediated leukogram variation [26], which could be attributed to changes in the adrenal function. It is known that high levels of cortisol influence the distribution of leukocytes in the blood, causing lymphopenia by inducing the migration of lymphocytes from the peripheral circulation [27] and neutrophilia [27, 28] by inhibiting the apoptosis of these cells [28].

Biochemical analysis revealed that the liver could be a target organ of the GCE toxicity, since the levels of alkaline phosphatase (AP) were significantly increased in the groups (male and female) treated with GCE at the highest dose (4 mg/kg, p.o.), and other biochemical parameters that could be related to liver toxicity were also altered in the plasma of the GCE-treated mice. AP is present in several tissues, but is particularly concentrated in the liver. Therefore, increased AP levels are related to liver injury rather than other reasons [24].

Generally, individual elevations of AP levels with no variations in ALT levels are related to hepatic cholestasis [29]. AP serum levels can be elevated due to the obstruction of the bile ducts, which is related to increased canalicular synthesis of AP with subsequent translocation to the hepatic sinusoid [30]. Therefore, cholestatic liver diseases are associated with increases in the synthesis and release of AP [31]. In this sense, we may suggest that GCE, at the highest dose, induced cholestatic hepatic injury in mice.

Additionally, other biochemical parameters, such as glucose, total cholesterol, LDL, triglycerides, total protein (in female mice), and albumin, were significantly affected in animals treated with GCE, mainly at 4 mg/kg. As the liver is the main organ related to metabolism (and the changed parameters are related to metabolism) [29, 32], we suggest that these variations could be related to liver damage. Nevertheless, no differences in the histopathological analysis of the liver were detected in the present study, indicating that the tissue damage was not sufficient to change its histological structure.

Hemostasis is directly associated with liver function, since several coagulation factors are synthesized by the liver [33]. In this sense, thrombocytopenia is a hematological change frequently found among patients with chronic liver disease [34]. Considering that the liver is the organ responsible for the activation of the coagulation cascade, and once damaged, it compromises the coagulation homeostasis [35], the variations in biochemical markers of hepatic function in mice treated with the highest GCE dose could be related to the thrombocytopenia [26] found in these animals.

The serological decrease in uric acid levels at the highest GCE dose and the discreet hyponatremia observed in the GCE 1 mg/kg-treated group may be related to the uricosuric action of plants from Poaceae family, such as *Cymbopogon martinii*. Interestingly, corn silk, *Zea mays* L. (Poaceae), presents uricosuric, diuretic, antilithogenic, and antiseptic properties [36] and is traditionally used worldwide for the treatment of edema, as well as for cystitis, gout, nephrolithiasis, nephritis, and prostatitis [37].

**Figure 9:**
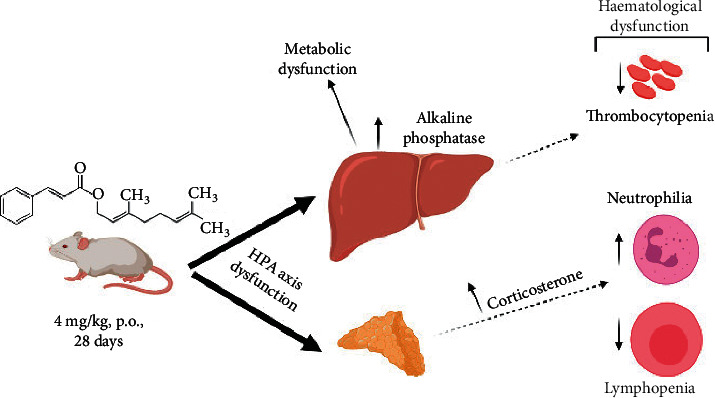
Diagram of the mechanisms involved in the toxic effect of GCE (4 mg/kg, p.o., 28 days) in mice. Dotted arrows indicate a possible effect (created with Biorender.com).

From the above discussion of the findings obtained in this study, it can be therefore suggested that the GCE, at 4 mg/kg, p.o., exerts its toxic effects on the animals by the following mechanisms: (i) liver injury (evidenced by the serological increase in the ALP enzyme), which leads to metabolic dysfunction (variations in serum glucose, proteins, and lipid levels) and, possibly, reduced production of coagulation factors, which are important for normal platelet function; (ii) adrenal hyperplasia, which might be related to GCE-induced dysfunction of the HPA axis, resulting in increased production of corticosterone, which impacts on the leukogram of the animals, causing lymphopenia and neutrophilia. These hypotheses are illustrated in Figure 9.

In conclusion, the GCE showed activity against *Candida albicans* and *Aspergillus niger* at very low concentrations as a very active compound for the tested fungi. Furthermore, this study comprises the first analysis on the toxicity of the geranyl cinnamate ester in experimental animals. The acute toxicity study demonstrated that the GCE can be classified into safety category 5, according to the OECD acute toxicity parameters. The study of repeated doses revealed that the lowest GCE dose is devoid of toxicity, which is extremely significant for the food industry, considering its application as a food preservative. Last, the biochemical and hematological variations observed in animals treated with GCE at the highest dose point to the liver as the target organ of potential GCE toxicity.

## Figures and Tables

**Table 1:** Concentration of geranyl cinnamate ester for the solid medium diffusion test.

Sample	Geranyl cinnamate volume (*μ*L)	Geranyl cinnamate concentration in the plate (*μ*L/mL)
a	50.00	10.00
b	25.00	5.00
c	12.50	2.50
d	6.25	1.25
e	3.12	0.62
f	1.56	0.31
g	0.78	0.16
i (control)	0	0

**Table 2:** Effect of the geranyl cinnamate ester (GCE) repeated-dose treatment (1, 2, and 4 mg/kg, p.o.) on female and male mice haemograms.

	Vehicle	GCE 1 mg/kg	GCE 2 mg/kg	GCE 4 mg/kg
*Male*				
WBC (×10³/mm³)	1.65 ± 0.37	2.16 ± 0.52	3.10 ± 0.38	2.94 ± 0.87
Hb (g/dL)	12.83 ± 1.34	11.78 ± 1.23	13.73 ± 1.34	12.88 ± 1.52
HCT (%)	38.20 ± 5.61	31.94 ± 4.67	41.63 ± 5.59	38.36 ± 4.86
MCV (*μ*m^3^)	48.50 ± 1.44	47.20 ± 0.20	47.20 ± 0.62	47.20 ± 0.44
MCH (pg)	16.15 ± 0.27	17.88 ± 1.21	16.23 ± 1.02	15.86 ± 0.16
MCHC (g/dL)	33.55 ± 1.08	37.96 ± 2.71	33.70 ± 1.75	33.84 ± 0.49
RDW (%)	16.75 ± 0.73	16.26 ± 0.17	16.73 ± 0.64	16.32 ± 0.19
Platelets (×10³/mm³)	373.80 ± 60.64	358.50 ± 60.33	475.50 ± 27.84	112.70 ± 5.55^*∗*^^/#/§§^
Erythrocytes (×10^6^/mm^3^)	6.76 ± 0.89	5.85 ± 1.52	6.93 ± 0.93	7.15 ± 0.34
Reticulocytes (%)	0.90 ± 0.08	1.20 ± 0.08	1.00 ± 0.08	0.80 ± 0.08^#^
Neutrophils	6.00 ± 0.86	7.00 ± 0.86	7.00 ± 0.86	10.00 ± 0.86^*∗*^
Lymphocytes	93.00 ± 1.03	91.00 ± 1.03	91.00 ± 1.03	88.00 ± 1.03^*∗*^
Monocytes	1.00 ± 0.29	0.00 ± 0.29	1.00 ± 0.29	0.00 ± 0.29

*Female*				
WBC (×10³/mm³)	2.13 ± 0.14	1.00 ± 0.20	1.95 ± 0.37	2.27 ± 0.60
Hb (g/dL)	13.60 ± 1.40	14.84 ± 0.49	15.26 ± 0.29	12.84 ± 0.71
HCT (%)	40.32 ± 5.03	45.18 ± 1.38	46.54 ± 1.05	36.72 ± 2.68
MCV (*μ*m^3^)	48.00 ± 0.57	47.20 ± 0.37	47.60 ± 0.24	47.20 ± 0.58
MCH (pg)	16.56 ± 0.70	15.44 ± 0.16	15.66 ± 0.10	16.58 ± 0.57
MCHC (g/dL)	34.28 ± 1.26	32.82 ± 0.28	32.82 ± 0.13	35.10 ± 0.86
RDW (%)	17.74 ± 1.37	16.04 ± 0.15	16.50 ± 0.21	16.12 ± 0.30
Platelets (×10³/mm³)	164.60 ± 44.89	137.60 ± 32.52	316.00 ± 115.5	253.00 ± 57.09
Erythrocytes (×10^6^/mm^3^)	6.71 ± 0.83	7.52 ± 0.23	7.75 ± 0.17	6.11 ± 0.44
Reticulocytes (%)	1.10 ± 0.11	1.50 ± 0.11	1.10 ± 0.11	1.00 ± 0.11^#^
Neutrophils	10.00 ± 0.47	11.00 ± 0.47	12.00 ± 0.47	12.00 ± 0.47
Lymphocytes	86.00 ± 0.57	88.00 ± 0.57	88.00 ± 0.57	86.00 ± 0.57
Monocytes	2.00 ± 0.48	3.00 ± 0.48	1.00 ± 0.48	3.00 ± 0.48

Data are expressed as mean + SEM. One-way ANOVA *post hoc* Bonferroni (*n* = 5 mice/group). ^*∗*^*p* < 0.05 different from vehicle-treated group; ^#^*p* < 0.05 different from GCE 1 mg/kg-treated group; ^§^*p* < 0.05 and ^§§^*p* < 0.01 different from GCE 2 mg/kg-treated group. Hb: hemoglobin, RDW: red cell distribution width, HCT: haematocrit, MCV: mean corpuscular volume, MCH: mean cell hemoglobin, MCHC: mean cell corpuscular hemoglobin concentration, WBC: white blood cell counts, erythrocyte count, reticulocytes, E: eosinophils, M: monocytes, N: neutrophils, L: lymphocytes, and platelet counts.

**Table 3:** Relative organs' weight (%) (brain, heart, thymus, spleen, adrenals, kidney, and liver) of the male and female mice treated with the geranyl cinnamate ester (GCE) at 1, 2, and 4 mg/kg (p.o.) for 28 days.

	Vehicle	GCE 1 mg/kg	GCE 2 mg/kg	GCE 4 mg/kg
*Male*				
Brain	1.18 ± 0.04	1.17 ± 0.01	1.16 ± 0.03	1.21 ± 0.02
Heart	0.39 ± 0.01	0.41 ± 0.01	0.43 ± 0.02	0.39 ± 0.01
Thymus	0.23 ± 0.01	0.21 ± 0.03	0.24 ± 0.01	0.23 ± 0.01
Spleen	0.29 ± 0.02	0.29 ± 0.01	0.31 ± 0.01	0.31 ± 0.01
Adrenals	0.020 ± 0.005	0.030 ± 0.004	0.030 ± 0.003	0.040 ± 0.004^*∗*^
Kidney	1.55 ± 0.02	1.60 ± 0.06	1.58 ± 0.07	1.69 ± 0.07
Liver	4.63 ± 0.26	4.84 ± 0.07	4.81 ± 0.07	4.73 ± 0.05

*Female*				
Brain	1.35 ± 0.07	1.36 ± 0.07	1.39 ± 0.0	1.46 ± 0.03
Heart	0.39 ± 0.02	0.37 ± 0.01	0.42 ± 0.02	0.41 ± 0.02
Thymus	0.33 ± 0.02	0.32 ± 0.02	0.28 ± 0.02	0.30 ± 0.03
Spleen	0.51 ± 0.08	0.38 ± 0.02	0.35 ± 0.02	0.40 ± 0.01
Adrenals	0.050 ± 0.007	0.060 ± 0.004	0.070 ± 0.008	0.080 ± 0.006
Kidney	1.44 ± 0.13	1.29 ± 0.11	1.27 ± 0.09	1.26 ± 0.05
Liver	4.71 ± 0.13	4.08 ± 0.18	4.20 ± 0.17	4.16 ± 0.23

Data are expressed as mean + SEM. One-way ANOVA *post hoc* Bonferroni. ^*∗*^*p* < 0.05 different from the vehicle-treated group (corn oil, 10 mg/mL, p.o.).

## Data Availability

Article data or supplementary data may be requested via email to the author Micheli Zanetti (eng.miche@unochapeco.edu.br) and will be shared with applicants.

## References

[1] Lucera A., Costa C., Conte A., Del Nobile M. A. (2012). Food applications of natural antimicrobial compounds. *Frontiers in Microbiology*.

[2] Pavithra P. S., Mehta A., Verma R. S. (2019). Essential oils: from prevention to treatment of skin cancer. *Drug Discovery Today*.

[3] Janjarasskul T., Tananuwong K., Kongpensook V., Tantratian S., Kokpol S. (2016). Shelf life extension of sponge cake by active packaging as an alternative to direct addition of chemical preservatives. *LWT—Food Science and Technology*.

[4] Gyawali R., Ibrahim S. A. (2012). Impact of plant derivatives on the growth of foodborne pathogens and the functionality of probiotics. *Applied Microbiology and Biotechnology*.

[5] Gyawali R., Hayek S. A., Ibrahim S. A. (2015). Plant extracts as antimicrobials in food products. *Handbook of Natural Antimicrobials for Food Safety and Quality*.

[6] Ozogul Y., Kuley E., Ucar Y., Ozogul F. (2015). Antimicrobial impacts of essential oils on food borne-pathogens. *Recent Patents on Food, Nutrition & Agriculture*.

[7] Solórzano-Santos F., Miranda-Novales M. G. (2012). Essential oils from aromatic herbs as antimicrobial agents. *Current Opinion in Biotechnology*.

[8] Ternus Z. M. (2015). Microbiological characterization of pure geraniol and comparison with bactericidal activity of the cinnamic acid in gram-positive and gram-negative bacteria. *Journal of Microbial & Biochemical Technology*.

[9] Frias D. F. R., Kozusny-Andreani D. I. (2008). Isolamento e identificação de fungos associados à dermatofitose e dermatomicose em cães. *Revista CES Medicina Veterinaria y Zootecnia*.

[10] Tang X., Shao Y.-L., Tang Y.-J., Zhou W.-W. (2018). Antifungal activity of essential oil compounds (geraniol and citral) and inhibitory mechanisms on grain pathogens (Aspergillus flavus and Aspergillus ochraceus). *Molecules*.

[11] Liu L., Hudgins W. R., Shack S., Yin M. Q., Samid D. (1995). Cinnamic acid: a natural product with potential use in cancer intervention. *International Journal of Cancer*.

[12] Tischer J. S., Possan H., Luiz J. (2019). Synthesis of eugenyl acetate through heterogeneous catalysis. *Journal of Essential Oil Research*.

[13] Zanetti M., Carniel T. K., Valério A. (2017). Synthesis of geranyl cinnamate by lipase-catalyzed reaction and its evaluation as an antimicrobial agent. *Journal of Chemical Technology & Biotechnology*.

[14] Organization for Economic Cooperation and Development (OECD) (2001). Guideline 423. Acute oral toxicity—acute toxic class method. https://ntp.niehs.nih.gov/iccvam/suppdocs/feddocs/oecd/oecd_gl423.pdf.

[15] Organization for Economic Cooperation and Development (OECD) (2008). Guideline 407. Repeated-dose 28-day oral toxicity study in rodents. https://www.oecd-ilibrary.org/environment/test-no-407-repeated-dose-28-day-oral-toxicity-study-in-rodents_9789264070684-en.

[16] Botelho M. A., Nogueira N. A. P., Bastos G. M. (2007). Antimicrobial activity of the essential oil from Lippia sidoides, carvacrol and thymol against oral pathogens. *Brazilian Journal of Medical and Biological Research*.

[17] Marcos-Arias C., Eraso E., Madariaga L., Quindós G. (2011). *In vitro* activities of natural products against oral *Candida* isolates from denture wearers. *BMC Complementary and Alternative Medicine*.

[18] Wang H., Yang Z., Ying G. (2018). Antifungal evaluation of plant essential oils and their major components against toxigenic fungi. *Industrial Crops and Products*.

[19] Cox S. D., Mann C. M., Markham J. L. (2000). The mode of antimicrobial action of the essential oil of *Melaleuca alternifolia* (tea tree oil). *Journal of Applied Microbiology*.

[20] Ghadirkhomi A., Safaeian L., Zolfaghari B., Agha Ghazvini M. R., Rezaei P. (2016). Evaluation of acute and sub-acute toxicity of *Pinus eldarica* bark extract in Wistar rats. *Avicenna journal of phytomedicine*.

[21] De Meijer V. E., Le H. D., Meisel J. A., Puder M. (2010). Repetitive orogastric gavage affects the phenotype of diet-induced obese mice. *Physiology & Behavior*.

[22] Hilaly J. E., Israili Z. H., Lyoussi B. (2004). Acute and chronic toxicological studies of Ajuga iva in experimental animals. *Journal of Ethnopharmacology*.

[23] Raza M., Shabanah O.-A., El-Hadiyah T. M. H., Al-Majed A. (2002). Effect of prolonged vigabatrin treatment on hematological and biochemical parameters in plasma, liver and kidney of Swiss albino mice. *Scientia Pharmaceutica*.

[24] Antonelli-Ushirobira T. M., Kaneshima E. N., Gabriel M., Audi E. A., Marques L. C., Mello J. C. P. (2010). Acute and subchronic toxicological evaluation of the semipurified extract of seeds of guaraná (Paullinia cupana) in rodents. *Food and Chemical Toxicology*.

[25] Guyton A. C., Hall J. E. (2012). *Tratado de Fisiologia Médica*.

[26] Thrall M. A. (2015). *Hematologia e Bioquímica Clínica Veterinária*.

[27] Cupps T. R., Fauci A. S. (1982). Corticosteroid-mediated immunoregulation in man. *Immunological Reviews*.

[28] Cameron R. G., Black P. N., Braan C., Browett P. J. (1996). A comparison of the effects of oral prednisone and inhaled beclomethasone dipropionate on circulating leukocytes. *Australian and New Zealand Journal of Medicine*.

[29] Kwo P. Y., Cohen S. M., Lim J. K. (2017). ACG clinical guideline: evaluation of abnormal liver chemistries. *American Journal of Gastroenterology*.

[30] Vroon D. H., Israili Z., Walker H. K., Hall W. D., Hurst J. W. (1990). Alkaline phosphatase and gamma glutamyltransferase. *Clinical Methods: The History, Physical, and Laboratory Examinations*.

[31] Xavier R. M., Dora J. M., Barros E. (2016). *Laboratório na Prática Clínica*.

[32] Betti A. H., Stein A. C., Dallegrave E. (2012). Acute and repeated-doses (28 days) toxicity study of *Hypericum polyanthemum* Klotzsch ex Reichardt (Guttiferare) in mice. *Food and Chemical Toxicology*.

[33] Sigal S., Mitchell O., Feldman D., Diakow M. (2016). The pathophysiology of thrombocytopenia in chronic liver disease. *Hepatic Medicine: Evidence and Research*.

[34] Qamar A. A., Grace N. D., Groszmann R. J. (2009). Incidence, prevalence, and clinical significance of abnormal hematologic indices in compensated cirrhosis. *Clinical Gastroenterology and Hepatology*.

[35] Kopec A. K., Joshi N., Luyendyk J. P. (2016). Role of hemostatic factors in hepatic injury and disease: animal models de-liver. *Journal of Thrombosis and Haemostasis*.

[36] Velazquez D. V. O., Xavier H. S., Batista J. E. M., de Castro-Chaves C. (2005). *Zea mays* L. extracts modify glomerular function and potassium urinary excretion in conscious rats. *Phytomedicine*.

[37] Grases F., March J. G., Ramis M., Costa-Bauzá A. (1993). The influence of Zea mays on urinary risk factors for kidney stones in rats. *Phytotherapy Research*.

